# Do Chinese viewers watch e-sports games for a different reason? Motivations, attitude, and team identification in predicting e-sports online spectatorship

**DOI:** 10.3389/fpsyg.2023.1234305

**Published:** 2023-10-13

**Authors:** Minlong Shi, Ruqin Ren

**Affiliations:** USC-SJTU Institute of Cultural and Creative Industry, Shanghai Jiao Tong University, Shanghai, China

**Keywords:** e-sports games, spectatorship motivation, the theory of reasoned action, team identification, satisfaction with past experience

## Abstract

**Introduction:**

Understanding factors that predict the intention of e-sports game online spectatorship has drawn a great deal of scholarly attention. Prior work on this topic has primarily focused on explaining the mediation mechanism between the spectator motivations and behavioral intention, such as attitude and subjective well-being, while overlooking the specific role of team identification and satisfaction with past experience, which were understudied in the previous studies but also prominent in the context of e-sports spectatorship. Besides, previous research was mainly conducted in a Western context; therefore, little was known about the online e-sports audience in China and their motivations to view e-sports games on live-streaming platforms.

**Methods:**

The current study thus aims to examine if and how these factors are related to the intention of watching e-sports games online by hierarchical regression and structural equation modeling.

**Results:**

Results from a survey of 452 Chinese e-sports online audiences suggested that three motivations (skill improvement, entertainment, and friends bonding), attitude towards e-sports game online spectatorship, and satisfaction with past experience were positively related to watching intention. These motivations and satisfaction also positively influenced attitude, while socialization opportunity (one of the motivations) negatively influenced attitude. Furthermore, team identification negatively moderated the relationship between satisfaction with past experience and behavioral intention. Also, attitude mediated the association between motivations, satisfaction, and behavioral intention.

**Discussion:**

In general, our study identifies the motivations that relate to Chinese e-sports viewers’ attitude as well as their watching intention, and underscores the role of satisfaction with past experience, attitude, and team identification in the context of e-sports. These findings contribute to deriving a holistic view of e-sports game online spectatorship.

## Introduction

Electronic sport (e-sport) is a video game-based competition between individual players or between teams, attracting millions of spectators to the host cities in person or online through live-streaming services (Kim and Kim, 2020). According to Newzoo (2022), e-sports would generate nearly $1.38 billion in revenues globally by the end of 2022, and China accounted for almost a third of worldwide esports revenues. However, in the last 3 years, along with the outbreak and recurrence of the COVID-19 pandemic, most e-sports game spectatorship gradually shifted from offline to online through live-streaming services, underscoring the importance of studying the formation of watching intention. After all, the key to the sustainable and healthy development of e-sports is to successfully attract and retain viewers, in which the watching intention is an important indicator to assess whether e-sports can attract and retain their viewers (Wang et al., 2021).

Within the existing literature of e-sports, the research on e-sports should be integrated with specific social environment and calls for more attention to the culture of e-sports environment experienced by e-sports fans. However, a majority of studies have focused on the e-sports industry in a Western context, while limited research exists explicitly on the e-sports industry in China, and even less specifically on Chinese e-sports fan’s watching behavior and watching intention. Notably, social acceptance levels of e-sports differ significantly across different cultures and it would influence the perceptions and attitudes of people towards e-sports engagement. For example, Cranmer et al. (2021) believed that the e-sports industry is better established in Asian markets than in Europe and the Americas. E-sports is an accepted and well-represented activity with a large follower base in South Korea. In their view, western cultures are often more associated with individualism, whereas cultures in South Korea tend to be collectively organized (Cranmer et al., 2021), which might explain the gap between Korea and the West in the social acceptance of e-sports. However, in China, where the culture also features collectivism, video games are metaphorized as “electronic heroin” (He and Cao, 2018). This trend did not show a decline until the last 20 years (He and Cao, 2018). Wong et al. (2021) investigated the perceptions and attitudes of teenagers and young adults toward e-sports engagement, revealing that e-sports was continually plagued by a poor social image in Hong Kong and, therefore, most parents disapproved of their children participating in e-sports.

Furthermore, several studies on e-sports engagement show limited understanding of Chinese e-sports fans’ watching intentions. For example, Wang et al. (2021) used a technology acceptance model to verify the influence of motivations on watching intention, such as social interaction, entertainment, and knowledge acquisition. However, this study ignored a key predictor variable of behavioral intention – attitude (Yan, 2014), as well as other relevant factors that matter in e-sports context, such as team identification and satisfaction with past experience. Similarly, a study conducted by Zhao et al. (2022) in China’s Henan Province confirmed the significant positive impact of perceived usefulness and perceived ease of use on the attitude toward e-sports, while overlooking the explaining power of motivation on attitude and behavioral intention.

As we design the research to more carefully investigate Chinese audiences’ e-sports watching intention, literature review found that empirical studies examining e-sports spectatorship so far still lacks consistent conclusions (Rietz and Hallmann, 2022). Some researchers seek to directly test the relationship between spectatorship motivations and watching intention. For example, a study conducted by Macey et al. (2022) found that knowledge acquisition, interaction with family and friends, and escapist motivation were positively associated with e-sports watching intention. However, Sjöblom et al. (2020) concluded that only vicarious achievement and novelty would positively predict future online consumption of e-sports. Given the mixed findings in the literature, we propose a research question as follows:

*RQ1:* In China, which motivation could influence the intention to watch e-sports games online directly?

Moreover, researchers explored the specific mediating mechanism between the spectatorship motivations and watching intention by testing different mediators. Different choices of mediating variables imply that scholars do not reach a consensus on how to understand the mechanism through which spectatorship motivations are associated with watching intention. Some of the mediator variables that have been tested include attitude (Xiao, 2020), flow experience, and subjective well-being (Kim and Kim, 2020). Although Xiao (2020) tried to explore the role of attitude to explain the relationship between spectatorship motivation and watching intention, he did not measure the indirect effect of spectatorship motivation on watching intention via attitude. Therefore, we propose a research question as follows:

*RQ2:* In the context of e-sports game online spectatorship, whether attitude can be confirmed as a mediator between motivation and watching intention? If any, how large is the indirect effect of spectatorship motivation on watching intention via attitude?

Besides, previous studies in traditional sports spectatorship have shown that factors, such as past watching experience and fans’ team identification, also significantly affect their attendance intention (Kaplanidou and Gibson, 2010; Lee et al., 2020).

The world of traditional sports constructs an experience in which a culture is formed where loyal fans are able to show support for their team through their words and actions (Brown et al., 2018). In this vein, the degree of a fan’s psychological connection with a team was operationalized by Wann (1994) as team identification, which has been a widely used concept in the context of spectator sports. Sporting events provide an exemplary platform to study team identification, as a collective identity that spectators develop with a team. As identity activation leads to behavioral involvement to engagement of action to express the collective identity (Ashmore et al., 2004), fans continue to attend sports games (Lee et al., 2020). In e-sports, the influence of professional teams on players and spectators is similar in many ways. However, little is known about the role of e-sports fans’ team identification on the formation of their watching intention. Moreover, past behavior has also been identified as a significant predictor of future behavior. Specifically, the significant effect of past behavior on future behavioral intentions has been corroborated in many fields, such as physical activity (Wang and Zhang, 2016), travel (Huang and Hsu, 2009), hockey games (Cunningham and Kwon, 2003) and the Senior Games (Kaplanidou and Gibson, 2010), while little is known about its role in the context of e-sports online spectatorship. Given these, we propose a research question as follows:

*RQ3:* In the context of e-sports game online spectatorship, how are past behavior and team identification associated with watching intention?

In summary, this study firstly seeks to validate the relationship between a set of e-sports spectatorship motivations and future watching intention in the context of Chinese e-sports environment. Moreover, while the direct link between motivation and intention seems plausible, it is also important to explore mechanisms that explain these relationships. Therefore, attitude would be served as a mediator to explain this relationship, as it is often regarded as a key predictor variable of behavioral intention. In addition to these well-studied factors in e-sports literature, this study would also explicate the role of past behavior and team identification in the formation of watching intention.

## Literature review

### The theory of reasoned action and the intention to watch e-sports games online

The theory of reasoned action (TRA, Fishbein and Ajzen, 1975) has been widely used as a model for the prediction of behavioral intentions and behavior in technology adoption. Considering TRA model’s long development history is often related to several other popular theoretical models, we will first briefly review them and discuss our choice of TRA model.

As e-sports online engagement requires esports consumers to continuously adopt and use new technology, including both hardware (i.e., smartphones and computers) and software (i.e., live-streaming software), a plethora of e-sports studies have applied the technology adoption theories (i.e., Technology Acceptance Model and Unified Theory of Acceptance and Use of Technology) as the theoretical framework to examine the antecedents and consequence associated with esports engagement (Jang and Byon, 2019; Jang et al., 2021; Meng-Lewis et al., 2022). For example, Meng-Lewis et al. (2022) integrated the Expectation Confirmation Model (ECM) with the Unified Theory of Acceptance and Use of Technology (UTAUT) to identify the determining factors predicting users’ intention to watch e-sports games on the live-streaming platform. To emphasize the hedonic value (intrinsic motivation) of technology users, Venkatesh et al. (2012) proposed UTAUT2, which incorporates three additional constructs into UTAUT: hedonic motivation, price value, and habit. UTAUT2 application studies involved users’ engagement in plethora of technologies such as gaming on mobile devices (Ramírez-Correa et al., 2019), social network sites (Herrero et al., 2017) and mobile tv (Wong et al., 2014). Majority of the UTAUT2 application studies examined user adoption decisions since they examined new to the market technologies in nascent stages of their product life cycle (Tamilmani et al., 2021). Similarly, Xu et al. (2023) used the technology acceptance model (TAM) to investigate how sports customers adopted over-the-top (OTT) services to consume sport content online. The TAM (Davis, 1989) is actually an adaptation of the theory of reasoned action (TRA) proposed by Fishbein and Ajzen (1975) to explain and predict the behaviors of people in a specific situation (Legris et al., 2003).

While TAM is widely recognized for its application in studying the adoption of online and mobile technologies, we have chosen to utilize the TRA as the theoretical framework for our study, motivated by two distinct reasons. On one hand, watching e-sports games through the live-streaming service may not be accepted as a process of embracing new technology in China. The first esports live streaming platform in China, Douyu TV, began operating in 2014 (Meng-Lewis et al., 2022), which means esports online spectatorship may not be perceived as novel by Chinese e-sports fans, but rather an integral component of their daily lives. Furthermore, Buabeng-Andoh (2018) indicated that TAM is less general than TRA to determine technology usage behavior. Therefore, our study would take the TRA as the theoretical framework to explore the antecedents associated with esports engagement.

The TRA theory posits that behavioral intentions are the immediate antecedents to the behavior (Madden et al., 1992), while one’s attitude toward certain behaviors and one’s perceived social pressure (i.e., subjective norms) are two of the main factors that directly correlate with one’s intention to perform an action (Ajzen and Fishbein, 1977, 1980; Xiao, 2020). The attitude toward the behavior refers to the degree to which a person has a favorable or unfavorable evaluation or appraisal of the behavior in question, and the subjective norm refers to the perceived social pressure to perform or not to perform the behavior (Ajzen, 1991). In the context of this study, we seek to define e-sports audience’s attitude towards e-sports game online spectatorship through the degree to which they have a favorable or unfavorable evaluation on their e-sports game online spectatorship. In the similar vein, subjective norms are defined as their perceived social pressure to watch e-sports games online.

Generally, the more favorable the attitude and subjective norm with respect to a behavior, the stronger should be an individual’s intention to perform the behavior under consideration (Ajzen, 1991). Over the past few decades, the predictive power of intention of actual behavior has been corroborated in many fields. Therefore, in the context of e-sports game online spectatorship, it seems plausible that those with strong watching intentions are more likely to view the e-sports tournaments through live-streaming services. In this vein, given the pivotal role of behavioral intention in the theory of reasoned action framework and its predictive power of actual behavior (Xiao, 2020), this study aims to explore the factors leading to behavioral intention.

In studies outside the domain of e-sports, Jeong et al. (2021) examined the process behind the decision of sports fans to attend sports matches at stadiums amid the pandemic. Their findings suggested that attitude and subjective norms would influence their attendance intention positively. A study conducted by Leung and Chen (2017) also showed that attitude and subjective norms could significantly influence consumers’ intentions to adopt mobile TV services.

Given the commonalities between watching mobile TV and watching e-sports games on an electronic device, we hypothesize that:

*H1:* Viewers’ attitude towards e-sports game online spectatorship is positively related to their future watching intention.

*H2:* Viewers’ subjective norms are positively related to future watching intention.

Tarkiainen and Sundqvist (2005) explored the relationships between subjective norms, attitudes, and intention to buy organic food by applying structural equation modeling. Not only did their results consolidate the predictive power of attitude in the consumers’ intention, but also confirmed the positive influence of subjective norms on the formation of attitude. Hence, the following hypothesis is posited.

*H3:* Viewers’ subjective norms are positively related to their attitude towards e-sports games online spectatorship.

### E-sports spectatorship motivations

Motivation is one of the most heavily studied constructs in sport-related research (Snelgrove et al., 2008). Due to the emergence of live-streaming platforms and e-sports games, an increasing number of studies have begun to call for work in the e-sports spectatorship motivations. Previous studies have primarily applied the uses and gratifications theory (UGT) to develop the motivation scale and examine the users’ motivations for selecting and consuming media in the context of traditional sports or e-sports (Trail and James, 2001; Hamari and Sjöblom, 2017; Sjöblom et al., 2017; Sjöblom and Hamari, 2017; Brown et al., 2018; Cabeza-Ramírez et al., 2020; Wulf et al., 2020). For example, Trail and James (2001) developed the Motivation Scale for Sport Consumption (MSSC) to measure the motivations behind sport spectator consumption behavior. The MSSC relies on a similar theoretical understanding as the UGT, in that it focuses on the gratification and experiences that sports consumption affords for spectators (Hamari and Sjöblom, 2017). The MSSC and UGT share many mutual aspects, such as an escapism from everyday life, acquiring information from the media content, being a fan, social interaction and so forth (Hamari and Sjöblom, 2017). In terms of the construct validity and reliability, the MSSC demonstrated the good psychometric properties (Trail and James, 2001), as illustrated in Tables 1, 2.

**Table 1:** The Cronbach’s alpha and AVE of the MSSC (Trail and James, 2001).

Factor	*α*	AVE
Achievement	0.89	0.74
Knowledge	0.80	0.59
Aesthetics	0.88	0.72
Drama	0.80	0.58
Escape	0.72	0.51
Family	0.68	0.48
Physical attraction	0.78	0.69
Physical skill	0.75	0.53
Social interaction	0.78	0.54

*α*, Cronbach’s alpha; AVE, Average variance extracted. The data was collected from 203 season ticket holders. All of the subscale AVE values exceeded 0.50, indicating that convergent validity was good.

**Table 2:** The correlations among factors in the MSSC (Trail and James, 2001).

	Ach	Kno	Aes	Dra	Esc	Fam	Att	Ski	Soc
Ach	1								
Kno	0.185	1							
Aes	0.339	0.463	1						
Dra	0.309	0.249	0.311	1					
Esc	0.656	0.023	0.329	0.319	1				
Fam	0.355	0.075	0.353	0.338	0.406	1			
Att	0.194	−0.014	0.147	−0.115	0.164	0.062	1		
Ski	0.465	0.257	0.622	0.411	0.455	0.630	0.160	1	
Soc	0.451	0.334	0.376	0.388	0.479	0.312	0.083	0.519	1

Ach, Achievement; Kno, Knowledge; Aes, Aesthetics; Dra, Drama; Esc, Escape; Fam, Family; Att, Physical attraction; Ski, Physical skill; Soc, Social interaction. None of the squared correlations exceeded the AVE values for any of the constructs, indicating that discriminant validity was good.

As one of the most widely applied scales for sports consumption, the MSSC has gone through rounds of revisions and has resulted in current variations, which commonly consist of 10 constructs, including vicarious achievement, aesthetics of sport, drama of sport, watching sports as a means to escape everyday life, knowledge acquisition related to the sport, admiring the skills of the athlete’s, social interaction with other spectators, physical attractiveness of the athletes, novelty of new players and teams, and the enjoyment of aggression and the aggressive behaviors the athletes exhibit (Trail and James, 2001; Hamari and Sjöblom, 2017). In a study that is more aligned with the goal of our current study, Xiao (2020) selected six motivators from the MSSC as behavioral beliefs to examine their correlation with the intention to watch e-sports. Also, Kim and Kim, (2020) applied the MSSC (Trail and James, 2001) in their e-sports spectatorship studies.

With the Internet has been a significant tool for sports consumption, professional sport teams’ Web sites became an important component of people’s sports consumption. Yet, little was know about the Internet users’ consumption motives for sport teams’ Web sites, as the established motivation scales (e.g., the MSSC) focused more on the traditional media. To this end, Seo and Green (2008) developed the Motivation Scale for Sport Online Consumption (MSSOC) to measure dimensions of motivation for traditional sport Internet users. Based on this study, Brown et al. (2018) applied the motivations from the MSSOC to compare the uses sought and gratifications obtained when consuming media related to e-sports and traditional sports.

Nevertheless, not every scholar agrees that traditional sports consumption motivations are the same as e-sports spectatorship motivations. To this end, Qian et al. (2020b) developed the Motivation Scale of Esports Spectatorship (MSES) by considering the innate links that connect esports with video gaming and traditional sport. The composite reliability (CR) and average variance extracted (AVE) values of the MSES are shown in Table 3, to validate the factor reliability, convergent validity, and discriminant validity of it. Although Qian et al. (2020b) did not mention the Cronbach’s alpha and correlation coefficient of the factors in the MSES in their study, they pointed out that its convergent validity and discriminant validity indices were all greater than the threshold values.

**Table 3:** The CR and AVE of the MSES (Qian et al., 2020b).

Construct	CR	AVE
Competitive nature	0.88	0.58
Socialization opportunity	0.95	0.78
Skill improvement	0.93	0.68
Friends bonding	0.93	0.72
Game knowledge	0.87	0.63
Skill appreciation	0.82	0.53
Entertaining nature	0.90	0.64
Dramatic nature	0.83	0.62
Competition excitement	0.95	0.83
Vicarious sensation	0.77	0.54

CR, Composite reliability; AVE, Average variance extracted. The data was collected from 638 e-sports fans.

Although the MSSC was treated as an adequate measure for esports spectating by Macey et al. (2022), they also highlighted that additional aspects specific to esports require further investigation. Consequently, esports online spectator motivation should be considered a hybrid of traditional sports spectatorship motivations and new media consumption motivations (Seo and Jung, 2016). In this case, our study attempts to combine the motivation research on e-sports and traditional sports. Grounded in previous studies, the motivations can be divided into three categories: (a) streamer-oriented attributes (b) technology-oriented attributes (c) individual motivations. Please see Appendix 1 for the dimensions and references of three motivation categories.

Streamer-oriented attributes refer to the personality traits and characteristics of streamers perceived by the audience, such as perceived social attractiveness (Kim H.-M. et al., 2023), perceived similarity (Kim H.-M. et al., 2023) and streamer skills (Kim and Kim, 2023). Technology-oriented attributes refer to the attributes of live-streaming platforms, live-streaming apps and over-the-top (OTT) services, such as ease of use (Wang et al., 2021), viewing quality (Xu et al., 2023) and stream quality (Qian et al., 2020a). Although these external elements do have a huge impact on e-sports viewing, this study aims to focus on the individual motivations for two reasons. Firstly, individual motivations are the intrapersonal or interpersonal elements (hedonic or internal) that influence people’s decision to consume e-sports (Qian et al., 2020a). Since this study aims to study the e-sports spectatorship from the perspective of Chinese e-sports fans, individual motivations are more in aligned with the goal of this study. Tang et al.’s (2022) study also indicated that esports spectatorship was driven significantly more by individual factors. In terms of the technology-oriented factors, the apps of different live-streaming platforms in China have almost the same features and functions, showing a trend of homogeneity. Essentially, these platforms are all easy to use and full of enjoyment. In addition to the technology factors, streamer-oriented factors are not as important as they are in the context of live game streaming, since there is no fixed streamer in the official e-sports event live-streaming room at all. Therefore, we believe that the differences in the individual motivations would be more influential than streamer-oriented attributes and technology-oriented attributes in this study.

In this case, we integrated the MSSC (Trail and James, 2001; Trail et al., 2003) with the MSES (Qian et al., 2020b) to achieve a new list of individual e-sports spectatorship motivations. In accordance with the goal of this study, we aim to incorporate a set of internal and intangible factors that motivate individuals to seek specific experiences in sports or esports spectatorship through the lens of their socio-psychological needs. Therefore, we selected seven individual motivations from the list, consistent with the context of online spectatorship. The seven motivations include: (1) skill improvement, (2) vicarious achievement, (3) knowledge acquisition, (4) escapism, (5) entertaining nature, (6) socialization opportunity, and (7) friends bonding, as Table 4 shows.

**Table 4:** Motivations for e-sports game online spectatorship.

Motivation	Definition
Skill improvement	The extent to which esports fans watching e-sports is to learn new skills, improve their own games, and imitate professionals (Qian et al., 2020b).
Vicarious achievement	The extent to which esports fans watching e-sports is to empathize and co-live with people and characters in media content, and in the sports context, with the achievements of teams and players (Hamari and Sjöblom, 2017).
Knowledge acquisition	The extent to which esports fans watching e-sports is to acquire an increase in knowledge about the game (Kim and Kim, 2020).
Escapism	The extent to which esports fans watching e-sports is to escape from day-to-day routines, and spectatorship provides a distraction from everyday activities (Hamari and Sjöblom, 2017).
Entertaining nature	The extent to which esports fans watching e-sports is to seek happiness and pleasure (Qian et al., 2020b).
Socialization opportunity	The extent to which esports fans watching e-sports is to interact with people online with similar interests and familiar identities, and obtain a feeling of belongingness, camaraderie, and social acceptance (Qian et al., 2020b).
Friends bonding	The extent to which esports fans bond with friends in reality to develop and maintain social relationship viawatching esports (Qian et al., 2020b).

Since motivations are at the core of e-sports spectatorship studies, a large body of research has directly related motivations with actual purposeful behaviors of watching e-sports. For example, Hamari and Sjöblom (2017) found that escapism, acquiring knowledge about the games being played positively predicted e-sport spectating frequency, and similarly, acquisition of knowledge, escapism, and friends were found to be positively associated with watching intention in another research (Macey et al., 2022). Brown et al. (2018) focused on comparing the motives when consuming media related to e-sports and traditional sports from a use sought and gratification perspective. Their results revealed that eSports participants sought out media for social sport, fanship, and Schwabism (a feeling of having superior knowledge) (Brown et al., 2018). In terms of a real-time strategy game, StarCraft II, motivations including vicarious achievement, skill of the athletes, and entertainment value were found to impact the game attendance frequency positively (Pizzo et al., 2018). Skill of the athletes might also attract viewers to watch e-sports games. As Qian et al. (2020b) confirmed before, skill improvement was the unique motive that emerged in the esports context. Given these existing studies and the predictive power of behavioral intention in actual behavior, we hypothesize that:

*H4:* Viewers’ e-sports game online spectatorship motivation is positively related to their future watching intention.

In Xiao’s research (2020), motivators seemed to reflect an individual’s behavioral outcome expectancies, and he applied the expectancy-value model to explain how motivators influence attitude formation. Their results uncovered a positive relationship between motivation (aesthetics, drama, and escapism) and attitude (Xiao, 2020). Likewise, a great deal of literature starts with the use and gratification theory (Katz et al., 1973), proving that in the context of e-sports spectatorship, those with different motives tend to meet their expectancies by watching e-sports online and subsequently fulfill both psychological and social needs (Hamari and Sjöblom, 2017; Pizzo et al., 2018; Kim and Kim, 2020). Thus, we assume that fans may develop a positive attitude toward watching e-sports games online if the behavior could meet their expectancies. A hypothesis is posited as follows:

*H5:* Viewers’ e-sports game online spectatorship motivation is positively related to their attitude towards e-sports game online spectatorship.

### Past behavior and satisfaction with past experience

Past behavior is typically the strongest predictor of future behavior (Ajzen, 1991; Wang and Zhang, 2016). Moreover, the significant effect of past behavior on future behavioral intentions has also been corroborated in many fields, such as physical activity (Wang and Zhang, 2016), travel (Huang and Hsu, 2009), hockey games (Cunningham and Kwon, 2003) and so on, while little is known about its role in the context of e-sports online spectatorship. When it comes to the measurement of past behavior, most researchers adopted a single item, such as “Including last season, how many _____ men’s hockey games have you attended (Cunningham and Kwon, 2003)?” This single-item measure is applicable in the context of travel and traditional sports spectatorship. However, it is not so appropriate for the context of e-sports game online spectatorship in this study. On one hand, e-sports is a broad notion that includes so many different types of games, and undoubtedly, there should be a big difference between these games. On the other hand, watching e-sports games online is quite different from offline spectatorship. The most obvious difference lies in that when watching e-sports games online, viewers are more likely to be influenced by other entertainment ways on their electronic devices. For example, Macey et al. (2022) found that e-sports spectating would positively influence gaming intention. That is to say, online viewers are likely to play games, use social media, or do other irrelevant things after watching a game for a while, which offers an inaccurate measurement in this construct.

Despite the operationalization of past experience being blurred and arguable, Huang and Hsu (2009) developed a new approach to solving the problem, with the measurement of overall satisfaction with past experience. In their studies, overall satisfaction refers to a subjective evaluation of all past travel experiences in the destination, and the results indicated that people are more likely to revisit a destination if they have had satisfactory past travel experiences with it (Huang and Hsu, 2009). Therefore, we decided to adopt the measurement of satisfaction with past experience to explain one’s past behavior (Huang and Hsu, 2009), instead of the previously used single item (Cunningham and Kwon, 2003; Kaplanidou and Gibson, 2010). To better align with the goal of this study, satisfaction with past experience was defined as one’s subjective evaluation of his past experiences in e-sports game online spectatorship. Given this, we hypothesize:

*H6:* Satisfaction with past watching experience is positively related to attitude.

*H7:* Satisfaction with past watching experience is positively related to future watching intention.

### Attitude as a mediator

In the TRA framework, attitude is determined by behavioral beliefs (Fishbein and Ajzen, 1975). Behavioral beliefs are one’s outcome expectancy of a behavior (Ajzen and Driver, 1991). Xiao (2020) believed that this expectancy-value logic proposed by Fishbein and Ajzen (1975) for TRA is akin to the need-gratification rationality proposed in U&G theory (Blumler, 1979) to explain media consumption behaviors and attitudes. Thus, in this study, spectatorship motivations can be seen as the equivalent to the behavioral outcome expectation, just as behavioral beliefs function in the TRA framework (Xiao, 2020). Lin (2014) applied the cognitive–affective–conative framework (CAC) (Fishbein and Ajzen, 1975) to explain the mediating role of attitude in the relationship between the beliefs and behavioral intention. He believed that the central theme in the TRA is that intention or willingness to perform a behavior (i.e., conative) is influenced by one’s attitude towards the behavior (i.e., affection). And a positive attitude towards the behavior is shaped by one’s beliefs and evaluations about performing the behavior (cognitive) (Lin, 2014). In the same vein, he found that attitude mediates the path between the effects of all gratifications (pastime, entertainment, relaxation, escape, and surveillance motives for local news) and intention to read citizen journalism news. In the context of sports, Kaplanidou and Gibson (2010) also revealed that there were mediation effects of attitudes between satisfaction and intentions to participate in the event again among the elderly. Given these, the following hypotheses on the mediating effects of attitudes are proposed:

*H8a:* Attitude mediates the relationship between e-sports game online spectatorship motivation and future watching intention.

*H8b:* Attitude mediates the relationship between satisfaction with past watching experience and future watching intention.

### The role of team identification

Team identification reflects the intensity of a spectator’s association, a collective identity, with his or her team (Tajfel, 1982; Lee et al., 2017). Within the sports context, identification significantly influences recurring behavior, such as watching games on TV and participating in second-screen conversations about their team (Cunningham and Eastin, 2017). Research conducted by Wann and Pierce (2003) indicated that a fan’s attachment to a team is an important predictor of numerous affective, cognitive, and behavioral reactions. Specifically, those with higher team identification are more likely to attend future games (Wann et al., 2001; Matsuoka et al., 2003). Furthermore, Lee et al. (2020) investigated the complexity of how spectators’ multiple identities influence their behavioral intention and posited that team identity directly affects their attendance intention. Notably, e-sports fans do not support every team. Instead, they are supporters of their favorite team during the spectatorship, and thus e-sports viewers are likely to own strong team identification and loyalty to specific teams. Given this, a hypothesis is proposed:

*H9:* Team identification is positively related to future watching intention.

Within the existing literature on traditional sports, researchers always classified sports fans into different groups based on the level of team identification in order to compare differences in their behaviors and feelings. For example, Bristow and Sebastian (2001) found significant differences between two fan groups in behavior and attitude to the Chicago Cubs baseball team, depending on the commitment of them. Wann et al. (1994) were able to show that highly identifying sport spectators reported an increase in positive emotions after watching a win, whereas low identifying viewers showed almost no emotional change. Schramm and Knoll (2017) used social identity theory to explain these differences that highly identifying sports fans view their team as being part of their social identity and therefore take a defeat of their team as a personal defeat and a win of their team as a personal victory resulting in respective mood. Furthermore, Matsuoka et al. (2003) assessed the interaction effects of team identification and satisfaction with facets of a game on intentions to attend future games, finding that the intentions of highly identified fans relative to low identified fans were less influenced by satisfaction. Given that e-sports fans also have varying levels of identification with different teams, similar to traditional sports fans, we hypothesize a moderation model.

*H10:* Team identification moderates the relationship between attitude and future watching intention.

*H11:* Team identification moderates the relationship between satisfaction with past watching experience and future watching intention.

**Figure 1:**
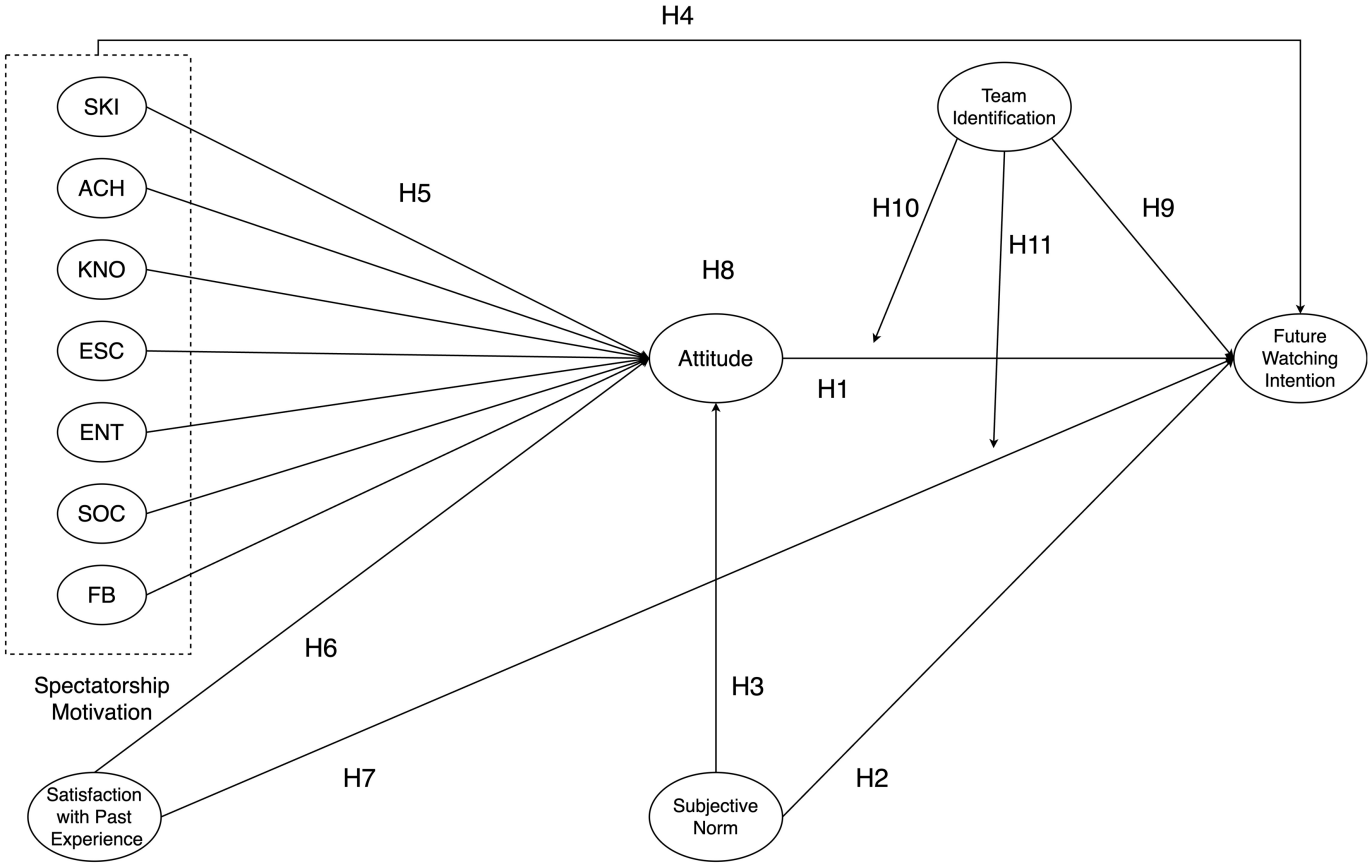
Conceptual model.

Based on the analysis, the full conceptual model is illustrated in Figure 1.

## Method

### Data collection and sample

This study collected data from October to November in 2022. In the first stage, a snowball sampling method was applied. We posted the online survey link in the game communities throughout social media platforms and also asked our friends to share the link with those who like to watch the e-sport games online. During the second stage, we recruited the respondents via an online survey platform, Sojump.^1^ All the participants were required to answer the screening question (whether they had watched e-sports games online before) on the first page of the online questionnaire, and 93 respondents who failed the screening questions were removed. Additionally, those who spent too much time (more than 500 s) or very little time (less than 100 s) completing the questionnaire were also removed. As a result, the final data set constituted 452 valid responses. The participants were rewarded with a monetary incentive (RMB12) after completing the survey.

Overall, the majority of participants were aged between 19 and 39 years (*n* = 411, 90.93%), mostly possessed a bachelor’s degree (*n* = 285, 63.05%), and were comprised of 183 (40.5%) females and 269 (59.5%) males. Compared to FPS (first-person shooter) games, participants obviously preferred watching e-sports tournaments based on MOBA (multiplayer online battle arena; *n* = 374, 82.74%) games. All the sample demographics are presented in Table 5.

**Table 5:** Sample demographics (*N* = 452).

Construct		*N*	%
Sex	Male	269	59.50
	Female	183	40.50
Age	12-	1	0.22
	13–15	5	1.11
	16–18	19	4.20
	19–22	100	22.12
	23–29	216	47.79
	30–39	95	21.02
	40–49	12	2.65
	50+	4	0.88
Education	Primary school	1	0.22
	Middle school	5	1.11
	High school	25	5.53
	Bachelor	285	63.05
	Master or Doctor	133	29.42
	Other	3	0.66
Type	MOBA	374	82.74
	FPS/TPS	65	14.38
	Other	13	2.88

### Measurement

After a screening question, qualified participants were asked to report their e-sports game online spectatorship motivation, attitude towards e-sports game online spectatorship, subjective norm, satisfaction with past experience, team identification and future online watching intention for the e-sports games. All measures were rated on a 7-point Likert scale ranging from 1 (strongly disagree) to 7 (strongly agree) unless otherwise stated.

### E-sports game online spectatorship motivation

The scale for e-sports game online spectatorship was adapted mainly from the MSSC (Trail and James, 2001) and the MSES (Qian et al., 2020b). The motivations include: skill improvement (α = 0.905, M = 5.65, SD = 1.02), vicarious achievement (α = 0.861, M = 5.88, SD = 1.12), knowledge acquisition (α = 0.759, M = 5.95, SD = 0.98), escapism (α = 0.849, M = 4.90, SD = 1.56), entertaining nature (α = 0.73, M = 6.00, SD = 0.91), socialization opportunity (α = 0.933, M = 4.63, SD = 1.60) and friends bonding (α = 0.899, M = 5.47, SD = 1.23). Twenty-five items were employed to measure seven motivations. Example items include “watching the e-sports game online helps me become a better player (skill improvement),” “I feel proud when my preferred team (or player) does well (vicarious achievement),” “I increase my knowledge about a game by watching e-sports game online (knowledge acquisition), “watching e-sports game online provides an escape for me from my day-to-day routine (escapism),” “I watch the e-sports game online because it is fun to watch (entertaining nature),” “I enjoy interacting with other fans online when watching the e-sports game online (socialization opportunity) and “watching the e-sports game online gives me a chance to bond with my friends (friends bonding).”

### Attitude towards e-sports game online spectatorship

The measurement of attitude followed the design in Xiao’s study (2020). Xiao measured the attitude of the e-sports audience toward watching e-sports using these four items. Specifically, attitude was assessed using four semantic differential scales in response to the following item: “For me, watching e-sports game online is ….” The four scale items were “Extremely bad–Extremely good,” “Extremely worthless——Extremely valuable,” “Extremely unpleasant——Extremely pleasant” and “Extremely Boring——Extremely Interesting” (Xiao, 2020). Participants provided responses on an interval scale ranging from 1 to 7, where 1 = Extremely bad and 7 = Extremely good, for example. The internal consistency of this measurement is good (α = 0.883, M = 5.89 SD = 0.93).

### Subjective norm

Three adapted items from Xiao (2020) were used to measure subjective norm. Examples include “I want to watch e-sports game online because my friends do so, and I want to belong to the group.” The internal consistency of this measurement is good (α = 0.830, M = 4.42, SD = 1.52).

### Satisfaction with past experience

Satisfaction with past experience was measured with three adapted items from the scale developed by Huang and Hsu (2009). Example items include “my overall evaluation on the past experience of watching e-sports game online is positive.” The reliability of this measurement is satisfactory (α = 0.849, M = 5.86, SD = 0.92).

### Team identification

Team identification was measured with three adapted items from the scale developed by Jeong et al. (2021). Examples include “when someone criticizes my favorite e-sports team, it feels like a personal insult.” The internal consistency of this measurement demonstrates strong reliability (α = 0.832, M = 4.25, SD = 1.51).

### Future watching intention

Two items from Leung and Chen (2017) were used to measure future watching intention. Example items include “I intend to watch e-sports game online when it becomes available.” The internal consistency of this measurement demonstrates strong reliability (α = 0.811, M = 5.62, SD = 1.25). All the measurement items and their descriptive statistics are shown in the Appendix 2.

### Control variables

Demographics (i.e., age, gender, and educational background) and the favorite type of e-sport game to watch was controlled for in this study.

### Data analysis

We performed the data analysis primarily using Mplus 8 software (version 8.3) and SPSS 24. Specifically, we utilized Mplus 8 for conducting structural equation modeling (SEM). To ensure the validity of the measures, we conducted confirmatory factor analyses (CFA) with maximum likelihood estimation using Mplus 8 software. We assessed the validity and reliability by examining Composite Reliability (CR), Average Variance Extracted (AVE), and the square root of AVE. In this study, we employed a customed calculator to calculate the CR and AVE values, which were obtained by calculating the standardized factor loadings of items. The functionality of this calculator has been verified by the authors.

The accepted threshold values for CR and AVE are 0.7 and 0.5, respectively. All item loadings should be higher than 0.5. Additionally, the square root of AVE must be greater than the correlation with any other constructs in the model to establish discriminant validity. As all the indicators were found to meet these standards, both validity and reliability were established (see Tables 6, 7 and Appendix 2).

**Table 6:** Convergent validity and reliability.

Construct	#Item	M	SD	α	AVE	CR
SKI	6	5.65	1.02	0.905	0.619	0.907
ACH	3	5.88	1.12	0.861	0.676	0.862
KNO	2	5.95	0.98	0.759	0.614	0.760
ESC	2	4.90	1.56	0.849	0.752	0.857
ENT	4	6.00	0.91	0.873	0.618	0.866
SOC	6	4.63	1.60	0.933	0.702	0.934
FB	5	5.47	1.23	0.899	0.652	0.903
ATT	4	5.89	0.93	0.883	0.659	0.885
SN	3	4.42	1.52	0.830	0.618	0.829
SAT	3	5.86	0.92	0.849	0.622	0.832
IDE	3	4.25	1.51	0.832	0.703	0.877
INT	2	5.62	1.25	0.811	0.689	0.816

M, Mean; SD, Standard deviation; *α*, Cronbach’s alpha; AVE, Average variance extracted; CR, Composite reliability; SKI, Skill improvement; ACH, Vicarious achievement; KNO, Knowledge acquisition; ESC, Escapism; ENT, Entertaining Nature; SOC, Socialization opportunity; FB, Friends bonding; IDE, Team identification; SAT, Satisfaction with past experience; INT, Future watching intention.

**Table 7:** Pearson correlations among variables.

	SKI	ACH	KNO	ESC	ENT	SOC	FB	ATT	SN	SAT	IDE	INT
SKI	**0.787**											
ACH	0.432	**0.822**										
KNO	0.643	0.393	**0.784**									
ESC	0.265	0.355	0.238	**0.867**								
ENT	0.564	0.489	0.550	0.393	**0.786**							
SOC	0.453	0.308	0.327	0.370	0.373	**0.838**						
FB	0.511	0.436	0.409	0.386	0.527	0.628	**0.807**					
ATT	0.601	0.466	0.508	0.382	0.686	0.455	0.599	**0.812**				
SN	0.391	0.264	0.309	0.358	0.313	0.593	0.520	0.430	**0.786**			
SAT	0.584	0.434	0.473	0.354	0.711	0.428	0.583	0.807	0.392	**0.789**		
IDE	0.288	0.422	0.174	0.340	0.215	0.500	0.336	0.363	0.497	0.309	**0.838**	
INT	0.473	0.392	0.332	0.328	0.499	0.419	0.524	0.671	0.363	0.633	0.406	**0.83**

The bolded data represent the square root of AVE. SKI, Skill improvement; ACH, Vicarious achievement; KNO, Knowledge acquisition; ESC, Escapism; ENT, Entertaining Nature; SOC, Socialization opportunity; FB, Friends bonding; IDE, Team identification; SAT, Satisfaction with past experience; INT, Future watching intention.

**Figure 2:**
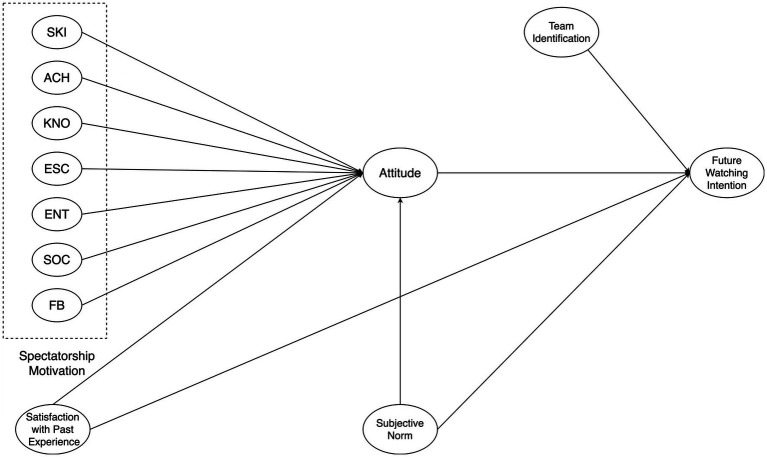
Hypothesized model examined in Mplus.

We also examined the fit of our hypothesized model (Figure 2) in Mplus 8. It was assessed with the comparative fit index (CFI), the root mean square error of approximation (RMSEA), and the standardized root mean squared residual (SRMR) (see Table 8). CFI value of 0.95 or higher, an RMSEA value of close to 0.06 or less, and an SRMR value of close to or less than 0.08 are indicative of good model fit (Hu and Bentler, 1999). In addition, chi-square statistics and Tucker-Lewis index (TLI) were also reported to suggest a satisfactory fit.

**Table 8:** The model fit.

χ^2^ / df	RMSEA	CFI	TLI	SRMR
1.921	0.045	0.946	0.939	0.040

SPSS 24 was applied to perform the hierarchical regression between the related factors and future watching intention. Furthermore, we used process 4.0 in SPSS to test the moderation and mediation effect of specific variables. By the calculation of SPSS 24, the Variance Inflation Factor (VIF) scores for all variables were between 1.063 and 3.626, which were well below the threshold of 5, indicating the absence of multicollinearity (Xiao, 2020).

## Results

### Hierarchical regression

In order to address *RQ1* and *H4*, we conducted hierarchical regression analysis to confirm the direct relationship between the relevant factors and future watching intention The results are shown in Table 9. We found that skill improvement (*β* = 0.180, *p* = 0.001), entertaining nature (*β* = 0.225, *p* < 0.001), and friends bonding (*β* = 0.242, *p* < 0.001) were positively related to the intention to watch e-sports games online. Thus, *RQ1* was addressed and *H4* was partially supported. The results also indicated that friends bonding was the strongest spectatorship motivation among e-sports fans in China.

**Table 9:** The hierarchical regression models.

	Model 1		Model 2		Model 3		Model 4	
	*β*	SE	*β*	SE	*β*	SE	*β*	SE
**Control**								
Age	0.151*	0.062	0.116**	0.052	0.077*	0.049	0.073*	0.047
Sex	0.028	0.118	0.047	0.096	0.067	0.089	0.055	0.086
Education	−0.084	0.085	−0.041	0.070	−0.020	0.065	−0.004	0.064
Type	−0.155**	0.124	−0.090*	0.101	−0.074*	0.094	−0.044	0.092
**Motivation**								
SKI			0.180***	0.067	0.095	0.063	0.068	0.062
ACH			0.085	0.052	0.012	0.051	0.008	0.050
KNO			−0.077	0.065	−0.057	0.060	−0.075	0.059
ESC			0.052	0.034	0.019	0.032	0.014	0.031
ENT			0.225***	0.071	0.059	0.075	0.003	0.074
SOC			0.053	0.040	−0.021	0.039	−0.004	0.039
FB			0.242***	0.056	0.170***	0.053	0.143**	0.052
**Other**								
IDE					0.174***	0.074	0.155***	0.037
SAT					0.398***	0.036	0.226***	0.084
**TRA**								
ATT							0.341***	0.085
SN							−0.042	0.037
Constant	5.821***	0.518	0.545	0.544	−0.25	0.515	−0.600	0.503
Adjusted R^2^	0.042		0.390		0.480		0.511	

*p < 0.05; **p < 0.01; ***p < 0.001. SKI, Skill improvement; ACH, Vicarious achievement; KNO, Knowledge acquisition; ESC, Escapism; ENT, Entertaining Nature; SOC, Socialization opportunity; FB, Friends Bonding; IDE, Team identification; SAT, Satisfaction with past experience; ATT, Attitude towards e-sports game online spectatorship; SN, Subjective norm.

### Path analysis

**Figure 3:**
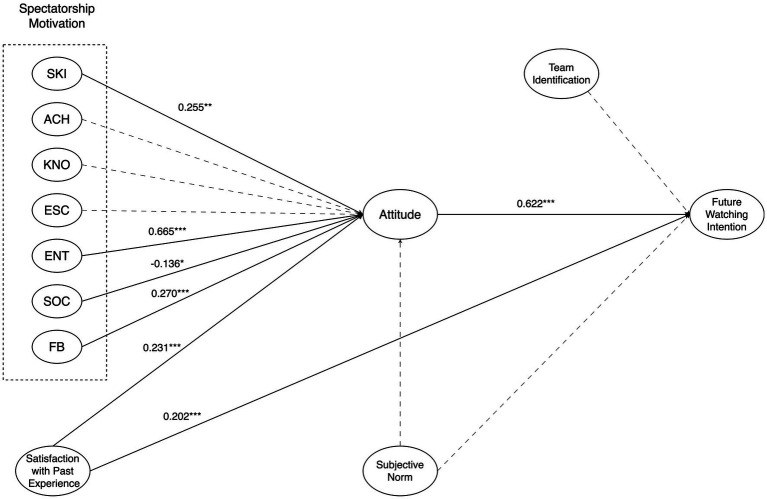
Summary of the path analysis.

To investigate *RQ3* and the related hypotheses, we examined the hypothesized relationships between constructs and reported standardized path coefficients, denoted as *β*. The model result is presented in Figure 3.

In our model, skill improvement (*β* = 0.255, *p* < 0.01), entertaining nature (*β* = 0.665, *p* < 0.001), friends bonding (*β* = 0.270, *p* < 0.001), and satisfaction with past experience (*β* = 0.231, *p* < 0.001) were positively associated with attitude towards e-sport game online spectatorship. Moreover, as expected, attitude (*β* = 0.622, *p* < 0.001) and satisfaction (*β* = 0.202, *p* < 0.001) could positively influence future watching intention.

Thus, *H1*, *H6*, and *H7* were confirmed, and *H5* was partially supported.

Furthermore, while the regression results showed a positive relationship between team identification (*β* = 0.155, *p* < 0.001) and watching intention, this path did not reach statistical significance in the structural equation model. Consequently, we concluded that *H9* was not fully supported. The details would be discussed as below.

### Mediation effect

Exploring *RQ2* and *H8*, we employed model 4 in Process 4.0 to examine the mediation effect of attitude. Based on our theoretical model, we hypothesized that attitude mediates the relationship between spectatorship motivators and watching intention, as well as the relationship between overall satisfaction and watching intention. The results confirmed the significant mediation role of attitude in the model (see Table 10). Thus, *RQ2* was addressed, *H8a* and *H8b* were supported.

**Table 10:** The mediation effect of attitude.

Outcome	Mediator	Predictor	*β*	95% Confidence intervals	
				LLCI	ULCI
Intention	Attitude	SKI	0.351	0.271	0.430
		ACH	0.284	0.216	0.354
		KNO	0.335	0.263	0.488
		ESC	0.230	0.163	0.302
		ENT	0.404	0.322	0.494
		SOC	0.268	0.206	0.331
		FB	0.314	0.243	0.387
		SAT	0.342	0.225	0.467

*β* refers to the completely standardized indirect effect; SKI, Skill improvement; ACH, Vicarious achievement; KNO, Knowledge acquisition; ESC, Escapism; ENT, Entertaining nature; SOC, Socialization opportunity; FB, Friends bonding; SAT, Satisfaction with past experience; ATT, Attitude towards e-sports game online spectatorship.

### Moderation effect

Model 15 in Process 4.0 was used to test the moderation effect of team identification. The results showed that team identification negatively moderated the relationship between satisfaction with past experience and future watching intention (*β* = −0.144, *p* < 0.01), while no significant moderation effect was found between attitude and intention (refer to Table 11). That is, high-identified individuals would persist in watching the games of their favorite team, despite their dissatisfaction with their past watching experience, while the low-identified individuals are influenced more by overall satisfaction with their past watching experience. Thus, *H11* was supported.

**Table 11:** The moderating effect of team identification.

	Model 1 attitude				Model 2 intention			
	*β*	SE	95%CI		*β*	SE	95%CI	
			Lower	Upper			Lower	Upper
Sex	0.083	0.052	−0.019	0.186	0.124	0.084	−0.041	0.289
Age	0.035	0.027	−0.019	0.089	0.083	0.045	−0.005	0.171
Education	−0.062	0.037	−0.135	0.012	−0.025	0.061	−0.144	0.095
Type	−0.15^**^	0.055	−0.258	−0.042	−0.147	0.089	−0.322	0.028
SAT	0.807^***^	0.028	0.751	0.862	0.294^***^	0.082	0.133	0.456
ATT					0.537^***^	0.084	0.371	0.702
IDE					0.163^***^	0.030	0.104	0.223
SAT*IDE					−0.144^**^	0.046	−0.234	−0.053
ATT*IDE					0.040	0.046	−0.050	0.131
F	175.0908^***^				55.122^***^			
R^2^	0.6625				0.5288			

*p < 0.05; **p < 0.01; ***p < 0.001. SAT, Satisfaction with past experience; ATT, Attitude towards e-sports game online spectatorship; IDE, Team identification.

## Discussion and conclusion

In general, this study identifies the motivations that relate to Chinese e-sports viewers’ attitude as well as their watching intention, and underscores the role of overall satisfaction, attitude and team identification in the context of e-sports. Findings related to these variables will be the foundation to help future scholars develop new lines of research to understand the factors behind e-sports fans’ online spectating behavior.

First, our findings imply that skill improvement, entertaining nature and friends bonding are defining motivations in the formation of attitude and behavioral intention among Chinese e-sports online audience, which differs from the previous studies mainly conducted in Western society (Hamari and Sjöblom, 2017; Kim and Kim 2020; Xiao, 2020; Macey et al., 2022). To our surprise, the motivation of socialization only negatively influences viewers’ attitude, while no influence is found on watching intention. These findings further suggest that social environment and cultural differences indeed matter in the domain of e-sports and should be considered more in the future.

Second, satisfaction with past watching experience is found to be as powerful as the three motivations (skill improvement, entertaining nature and friends bonding) in influencing the attitude and the watching intention, though often ignored in prior literature on e-sports games.

Third, our study introduces the moderating role of team identification on the relationship between satisfaction and behavioral intention. In this case, exploration of the mechanism of team identification provides a deeper insight into understanding how e-sports fandom is relevant in shaping viewers’ behavioral intention. Also, the mediating role of attitude in the formation of watching intention is confirmed. Below, we will discuss these findings in detail.

### Key spectatorship motivations among Chinese e-sports fans

This study contributes to e-sports spectatorship studies by revealing the significant motivations that are associated with attitude and watching intention. Previous studies were mainly conducted in the Western and ignored some defining characteristics of e-sports game spectatorship. This study goes beyond it by creating a new list of spectatorship motivations, consistent with the context of e-sports game online spectatorship. It indicates the powerful influence of skill improvement, entertaining nature and friends bonding on Chinese e-sports fans’ attitude and watching intention.

Skill improvement is the unique motive that emerges in the e-sports context (Qian et al., 2020b). Furthermore, e-sports gameplayers are always considered to be potential viewers of e-sports events (Tang et al., 2022), therefore it is plausible that these e-sports spectators tend to watch others play to improve their own gameplay performance (Seo and Jung, 2016; Jang and Byon, 2019; Qian et al., 2020b; Tang et al., 2022).

In addition, our study shows that the entertaining nature of e-sports game online spectatorship is associated with attitude and future watching intention, which is in line with previous work showing that esports is now considered a popular form of spectator entertainment (Brown et al., 2018; Qian et al., 2020b,c). One plausible explanation is that entertainment has a positive effect on creating pleasant emotions (Tan, 2008) and emotional gratification (Bartsch, 2012), which in turn elicits the favorable attitude and behavioral intentions (Eckler and Bolls, 2011; Foroughi et al., 2019).

Besides, it is also worth noting how esports might exert a positive impact on an existing social relationship or established friendship (Pizzo et al., 2018; Qian et al., 2020b), as our results show that friends bonding always positively influences watching intention, especially in China. On one hand, e-sports online spectatorship, as the digital counterpart of live events, provides bonding opportunities and social gratification similar to traditional sport spectatorship for e-sports fans (Qian et al., 2020b). On the other hand, because of the nature of e-sports and the relatively novelty of this phenomenon (Macey et al., 2022), the primary influencers when people first started watching esports were their close friends (Qian et al., 2020b). Therefore, it is common that sharing with close friends about e-sports games in China not only creates an environment that extends beyond mere socialization (Funk et al., 2004), but also treated as a means of relationship strengthening (Kissane and Winslow, 2016), particularly with friends in their social circle.

Prior work on this topic found that escapism (Hamari and Sjöblom, 2017) and knowledge acquisition (Hamari and Sjöblom, 2017; Tang et al., 2022) positively predict e-sports spectatorship. Besides, escapism was found to be positively related to attitude toward watching e-sports (Xiao, 2020). These findings are entirely different from the conclusions of this study. In our study, e-sports fans in China are motivated to watch games by their competence, entertainment and social motivations, as opposed to their informational motivations (Cabeza-Ramírez et al., 2020) or escapism (Hamari and Sjöblom, 2017). The significant differences in these motivations may perhaps explain why the e-sports industry has been able to rapidly grow in China, as watching and discussing e-sports games have become a part of people’s daily entertainment and social interaction.

### Satisfaction with past watching experience

In addition to extending the studies in spectatorship motivation, this study also seeks to understand the role of past behavior. Our study reveals a stable positive relationship between satisfaction and both attitude and behavioral intention.

Actually, rich literature in the broad leisure industry (such as tourism) has identified the critical role of past satisfaction (Petrick et al., 2001; Huang and Hsu, 2009; Huang et al., 2015), though it is understudied in the e-sports context. The underlying assumption of these studies is if an experience has a positive effect on an individual, they are more likely to repeat the activity (Petrick et al., 2001). Therefore, it is plausible that e-sports fans may develop positive emotions associated with e-sports online spectatorship based on their prior experience, as rewatching behavior resembles a rational planned behavior, similar to what is observed in tourism revisiting behavior (Huang and Hsu, 2009).

In general, the study contributed to the understanding of past watching experience’s role in future watching behavior by including satisfaction as a component of the construct.

### Explicating the role of attitude and team identification in e-sports spectatorship

This study also highlights attitude as a mediating mechanism through which spectating motivations and satisfaction with past experience are associated with future watching intention. Previous studies on e-sports spectatorship mainly focused on simply verifying the relationship between motivation, attitude and watching intention, while no one seeks to test the mediation effect of attitude and confirm the mediating role of it in the context of e-sports online spectatorship. Our study is one of the first studies to confirm the mediating role of attitude in the context of e-sports online spectatorship. Kim M. et al. (2023) explained the mediating power of attitude on spectator revisit intentions of Paralympics with the concept of sequential relationships among cognitive, affective, and behavioral components, which is common in the tourism literature (Kwon and Vogt, 2010; Wang et al., 2020). In the context of reading citizen journalism news, Lin (2014) also applied the cognitive–affective–conative framework (CAC) (Fishbein and Ajzen, 1975) to explain the mediating role of attitude in the relationship between the beliefs and behavioral intention. As motivation could be considered as a cognitive component (Kim M. et al., 2023), it is plausible that affective attitude works as a mediator on the effects of cognitive component (motivation) on behavioral intention (Wang et al., 2020).

**Figure 4:**
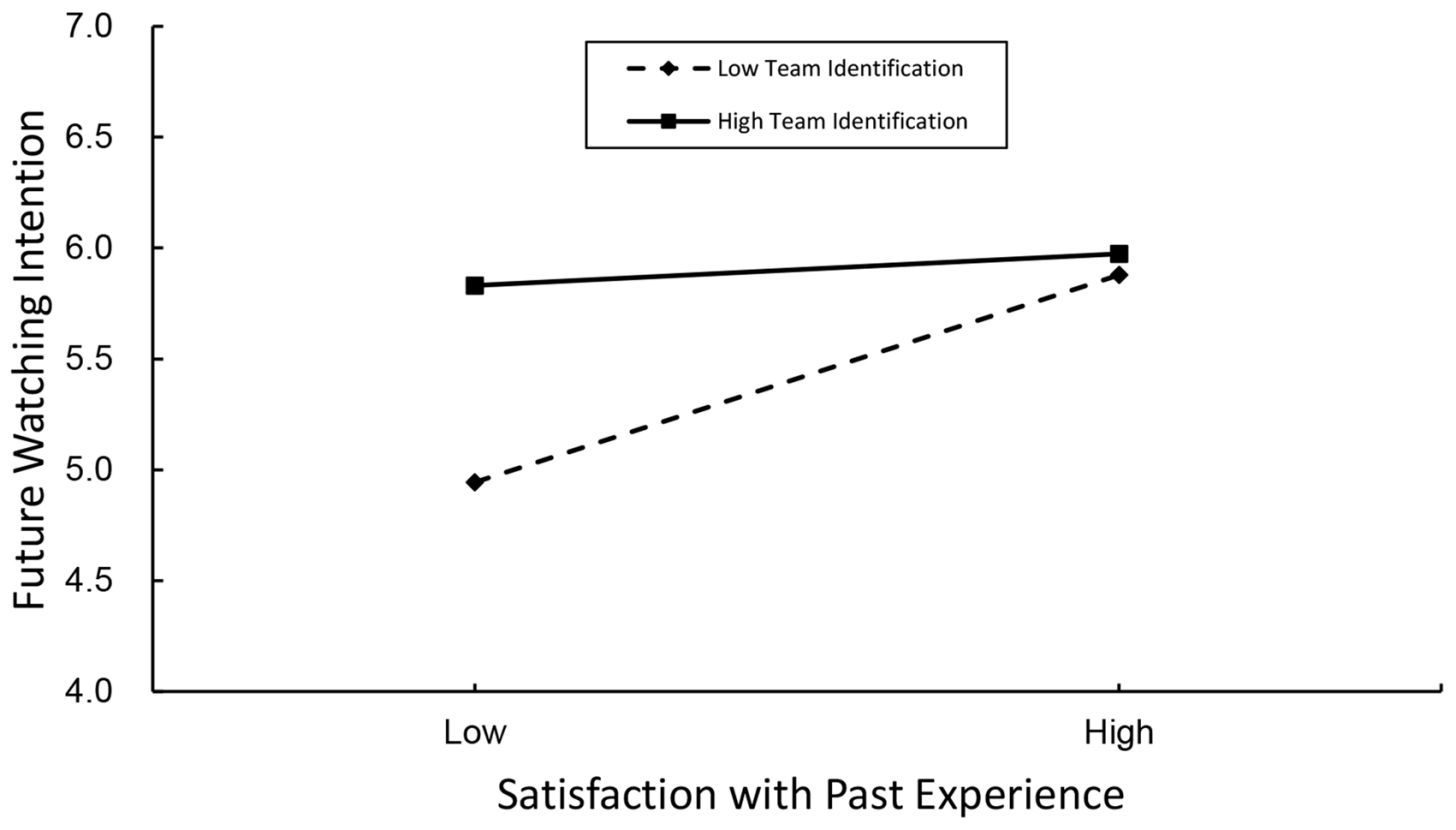
Interactive effect of team identification and satisfaction with past experience on future watching intention.

In our study, we also found that the interaction of team identification and satisfaction with past watching experience is a significant predictor of future watching intention. As can be seen in Figure 4, both high-identified e-sports viewers and low-identified fans expressed lower levels of intention to watch e-sports games online in the future when they were less satisfied with their past watching experience. However, such reduction in future watching intention was much more pronounced in the case of low-identified viewers than high-identified viewers. Specifically, those low-identified individuals would be influenced heavily by satisfaction with past watching experience. One possible explanation is that team identification is always strongly associated with psychological factors such as trust (Wu et al., 2012), fan loyalty (Bodet and Bernache-Assollant, 2011), and a sense of belonging (Heere and James, 2007), thus influences the perception high-identified individuals have of their own behaviors. In this vein, it is reasonable to expect that they would persist in watching the games of their favorite team, despite their dissatisfaction with their past watching experience. As for these low-identified ones, overall satisfaction plays a more critical role in their future watching intention, as a result of a lack of perceived psychological connection with a specific team. This finding is consistent with previous studies conducted in the domain of traditional sports (Bristow and Sebastian, 2001; Matsuoka et al., 2003), underscoring the significant influence of team identification on sports or e-sports fans’ intentions and behaviors.

While previous research suggested that team identification positively influences future intention (Wakefield, 1995; Matsuoka et al., 2003; Trail et al., 2003), our study did not arrive at the same conclusion. This discrepancy may be attributed to differences in our measurement of future watching intention, which was not limited to watching only the games of the e-sports fan’s favorite team, unlike previous studies that measured it through the name of a specific team (Wakefield, 1995; Matsuoka et al., 2003). Additionally, the development of team identification in esports is unique and always in a more fluid and dynamic way (Qian et al., 2023). This implies that investigating its precise generative mechanisms within the context of e-sports could be a promising direction for future research.”

### Practical implication

Our study also provides some practical implications. First, the motivations defined in our study suggest a need for live-streaming platforms and e-sports game organizers to devote more attention to the practical, entertaining, and social value of e-sports games. E-sports practitioners should implement specific mechanisms to help viewers better understand and learn from the gameplay of professional players, such as live replays and in-depth gameplay analysis during live-streaming. Given the popularity of e-sports games as a social activity among friends, e-sports game organizers should schedule more competitive games to encourage discussion among viewers. Second, the mediating role of attitude highlights the importance of cultivating a positive attitude towards e-sports game online spectatorship. E-sports practitioners should focus on creating a favorable attitude among spectators. Third, considering the moderating role of team identification, e-sports practitioners should aim to enhance overall satisfaction for low-identified viewers by improving live-streaming quality and reducing game pauses. On the other hand, e-sports clubs can foster loyal fans with high team identification by recruiting star players or enhancing their team’s performance.

### Limitation

However, this study has certain limitations. Firstly, the sample composition is limited, and the data analysis is cross-sectional, which only reveals the interrelationships between constructs. Future studies could employ longitudinal research to examine the causal relationships between these variables.

Secondly, this study did not impose restrictions on the types of e-sports games. However, each game has its defining characteristics, and preferences for specific game types would influence spectating behaviors (Tang et al., 2022). Therefore, future research could focus on a specific game, such as League of Legends (MOBA) and VALORANT (FPS).

Third, the identification scale in this study solely focuses on team identification, while player identification and e-sports identification may also be significant factors in e-sports game online spectatorship. Future research should consider including them in the context of e-sports and exploring the e-sports fandom culture further.

Lastly, gender differences exist in this male-dominated domain, particularly in terms of unique e-sports consumption motivations (Yu et al., 2022), but this study did not emphasize them. Therefore, scholars should make more effort to explore possible gender differences in the context of e-sports.

## Data availability statement

The raw data supporting the conclusions of this article will be made available by the authors, without undue reservation.

## Ethics statement

The studies involving humans were approved by USC-SJTU Institute of Cultural and Creative Industry Ethics Board. The studies were conducted in accordance with the local legislation and institutional requirements. The participants provided their written informed consent to participate in this study.

## Author contributions

MS contributed by idea generation, data collection, data analysis, and writing the manuscript. RR contributed by providing critical feedback and assistance in every part of the study, and suggestions to the initial and revised drafts. All authors contributed to the article and approved the submitted version.

## Footnotes

^1^ http://www.wjx.cn
