# Supplementary material for: Do Chinese viewers watch e-sports games for a different reason? Motivations, attitude, and team identification in predicting e-sports online spectatorship
Source: Front Psychol. 2023 Oct 13;14:1234305. doi: 10.3389/fpsyg.2023.1234305 (PMC10613109; doi:10.3389/fpsyg.2023.1234305)
Supplement: Supplementary file 1 [file Table_1.docx]

**Appendix 1:**

Motivation category.

| Motivation category | Dimensions | References |
| --- | --- | --- |
| Streamer-oriented | 1.Broadcaster appeal/Broadcaster attractiveness | Xu et al., 2021; Xu et al., 2021b  Ma et al., 2021; Hamari and Sjöblom, 2017; Huettermann et al., 2023  Qian et al., 2020a; Qian et al., 2020c  Qian et al., 2020a; Qian et al., 2020c  Kim et al., 2023a  Kim et al., 2023a |
|  | 2.Physical attractiveness |  |
|  | 3.Streamer traits |  |
|  | 4.Commentary features  5.Perceived social attractiveness  6.Perceived similarity |  |
| Technology-oriented | 1.Medium appeal  2.Stream quality/Viewing quality  3.Chat room  4.Virtual rewards  5.Donate features  6.Equipment cost | Xu et al., 2021;  Qian et al., 2020a; Qian et al., 2020c; Xu et al., 2023  Qian et al., 2020a; Qian et al., 2020c; Tang et al., 2022  Qian et al., 2020a; Qian et al., 2020c  Tang et al., 2022  Tang et al., 2022  Qian et al., 2020b  Kim and Kim, 2023  Kim and Kim, 2023; Xu et al., 2023  Kim and Kim, 2023; Xu et al., 2023  Kim and Kim, 2023; Xu et al., 2023  Xu et al., 2023  Kim and Kim, 2023  Kim and Kim, 2023  Xu et al., 2023  Qian et al., 2020a; Sjöblom and Hamari, 2017; Ma et al., 2021; Hamari and Sjöblom, 2017; Sjöblom et al., 2020; Kim and Kim, 2020b; Tang et al., 2022; Xiao, 2020; Qian et al., 2020b; Huettermann et al., 2023  Sjöblom et al., 2017; Hilvert-Bruce et al., 2018; Cabeza-Ramírez et al., 2020  Qian et al., 2020a; Ma et al., 2021; Hamari and Sjöblom, 2017; Sjöblom et al., 2020; Kim and Kim, 2020b; Qian et al., 2020b; Kim and Kim, 2023  Qian et al., 2020a; Qian et al., 2020b  Qian et al., 2020a; Hilvert-Bruce et al., 2018; Cabeza-Ramírez et al., 2020; Wulf et al., 2020; Tang et al., 2022; Qian et al., 2020b  Sjöblom et al., 2017; Sjöblom and Hamari, 2017  Qian et al., 2020a; Sjöblom and Hamari, 2017; Sjöblom et al., 2017; Ma et al., 2021; Hilvert-Bruce et al., 2018; Cabeza-Ramírez et al., 2020; Hamari and Sjöblom, 2017; Sjöblom et al., 2020; Kim and Kim, 2020b; Xiao, 2020; Qian et al., 2020b; Kim and Kim, 2023; Huettermann et al., 2023  Hilvert-Bruce et al., 2018; Tang et al., 2022; Kim and Kim, 2023  Sjöblom and Hamari, 2017; Sjöblom et al., 2017  Qian et al., 2020a; Sjöblom et al., 2017; Qian et al., 2020b  Sjöblom and Hamari, 2017; Cabeza-Ramírez et al., 2020  Ma et al., 2021; Hamari and Sjöblom, 2017; Sjöblom et al., 2020; Kim and Kim, 2020b; Xiao, 2020; Huettermann et al., 2023  Ma et al., 2021; Hamari and Sjöblom, 2017; Sjöblom et al., 2020; Xiao, 2020; Huettermann et al., 2023  Ma et al., 2021; Wulf et al., 2020; Hamari and Sjöblom, 2017; Sjöblom et al., 2020; Kim and Kim, 2020b; Xiao, 2020; Qian et al., 2020b; Huettermann et al., 2023  Ma et al., 2021; Hamari and Sjöblom, 2017; Sjöblom et al., 2020; Kim and Kim, 2020b; Tang et al., 2022; Xiao, 2020; Kim and Kim, 2023; Huettermann et al., 2023  Ma et al., 2021; Hamari and Sjöblom, 2017; Sjöblom et al., 2020; Huettermann et al., 2023  Ma et al., 2021; Hamari and Sjöblom, 2017; Sjöblom et al., 2020; Huettermann et al., 2023  Wulf et al., 2020; Kim and Kim, 2020b; Qian et al., 2020b; Kim and Kim, 2023  Wulf et al., 2020; Tang et al., 2022  Tang et al., 2022 |
| Individual | 7.Vicarious sensation  8.Aesthetic quality  9. Convenience  10.Information quality/Content quality  11.Perceived usefulness  12. Ease of use  13.Technology and functional quality  14.Security quality  15. Cost of subscription  1.Game knowledge  2.Information seeking  3.Skill appreciation  4.Competitive nature |  |
|  | 5.Entertaining nature/Enjoyment  6.Affective  7.Socialization/Social integrative/Social integration  8.Sense of community/Group affiliation/Shared emotional connection  9.Personal integrative  10.Skill improvement/Learning to play  11.Tension release  12.Vacrious achievement  13.Aesthetics  14.Drama/Suspense  15.Escape  16.Enjoyment of aggression  17.Novelty  18.Friendship/Friends bonding  19.Respectful interest/Sports fandom  20.Preference for game types |  |

**Appendix 2:**

Measurement items.

| Construct | Item | SFL | M | SD | Source |
| --- | --- | --- | --- | --- | --- |
| Skill Improvement | SKI 1 Watching the esports game online helps me become a better player.  SKI 2 I get to learn something new from some of the best players.  SKI 3 It would give me a better idea on how to win the game if I play.  SKI 4 I can improve my game by looking at techniques and strategies used by the experts.  SKI 5 It gives me a deeper understanding of what’s possible when I play.  SKI 6 It improves my own play by getting ideas from professional players. | 0.766  0.808  0.824  0.834  0.664  0.812 | 5.61  5.90  5.62  5.56  5.70  5.55 | 1.195  1.202  1.237  1.293  1.179  1.308 | (Qian et al., 2020b) |
| Vicarious Achievement | ACH 1 I feel proud when my preferred team (or player) does well.  ACH 2 I feel a personal sense of achievement when my preferred team (or player) does well.  ACH 3 I feel like I have won when my preferred team (or player) wins. | 0.845  0.820  0.801 | 6.02  5.73  5.90 | 1.217  1.329  1.249 | (Kim and Kim, 2020b) |
| Knowledge Acquisition | KN0 1 I increase my knowledge about a game by watching e-sports game online.  KN0 2 I can learn about the technical aspects of a game by watching e-sport game online. | 0.728  0.835 | 5.91  5.98 | 1.136  1.038 | (Kim and Kim, 2020b) |
| Escapism | ESC 1 Watching e-sport game online provides an escape for me from my day-to-day routine.  ESC 2 Watching e-sport game online provides a diversion from “life’s little problems” for me. | 0.777  0.949 | 4.69  5.11 | 1.715  1.636 | (Kim and Kim, 2020b) |
| Entertaining Nature | ENT 1 I watch the esports game online because it is fun to watch  ENT 2 I watch the esports game online because I want to have fun  ENT 3 I watch the esports game online because it is enjoyable to watch  ENT 4 It is a lot of fun to watch the esports game online | 0.770  0.802  0.802  0.771 | 5.92  6.01  5.99  6.10 | 1.099  1.065  1.130  0.991 | (Qian et al., 2020b) |
| Socialization Opportunity | SOC 1 I enjoy interacting with other fans online when watching the esports game online  SOC 2 It allows me to meet other people online with similar interests to mine  SOC 3 It provides an online social outlet when watching the esports game online  SOC 4 I can connect with other esports fans and be part of the online community  SOC 5 I enjoy interacting with streamers online and getting to know them  SOC 6 I can interact with other spectators online and get a sense of camaraderie | 0.818  0.836  0.842  0.873  0.805  0.851 | 4.29  4.85  4.94  4.64  4.55  4.51 | 1.886  1.819  1.778  1.843  1.916  1.873 | (Qian et al., 2020b) |
| Friends Bonding | FB 1 Watching the esports game online gives me a chance to bond with my friends  FB 2 I enjoy sharing the experience of watching the esports game online with friends  FB 3 I can have a good time with friends while watching the esports game online  FB 4 Watching the esports game online creates bonding moments that people can carry with them  FB 5 I enjoy watching esports games online with friends in a social setting | 0.825  0.830  0.868  0.779  0.728 | 5.32  5.49  5.61  5.67  5.25 | 1.494  1.446  1.355  1.297  1.658 | (Qian et al., 2020b) |
| Attitude towards  e-sports game online spectatorship | ATT 1 For me, watching e-sport game online is ...（Extremely bad——Extremely good）  ATT 2 For me, watching e-sport game online is ...（Extremely worthless——Extremely valuable）  ATT 3 For me, watching e-sport game online is ...（Extremely unpleasant——Extremely pleasant）  ATT 4 For me, watching e-sport game online is ...（Extremely Boring——Extremely Interesting） | 0.841  0.775  0.839  0.789 | 5.92  5.64  6.03  5.96 | 1.051  1.149  1.008  1.097 | (Xiao, 2020) |
| Subjective Norm | SN 1 I want to watch e-sport game online because my friends do so, and I want to belong to the group  SN 2 Watching e-sport game online reflects my personality to other people  SN 3 According to people who are important to me, I should watch e-sport game online | 0.744  0.818  0.795 | 4.38  4.65  4.24 | 1.835  1.658  1.769 | (Xiao, 2020) |
| Satisfaction with past experience of watching e-sport game online | SAT 1 My overall evaluation on the past experience of watching e-sport game online is positive  SAT 2 I am satisfied with my past experience of watching e-sport game online.  SAT 3 I am pleased with my past experience of watching e-sport game online. | 0.783  0.811  0.772 | 5.85  5.92  5.81 | 1.058  1.006  1.068 | (Huang and Hsu, 2009) |
| Team Identification | IDE 1 When someone criticizes my favorite e-sport team, it feels like a personal insult  IDE 2 When my favorite e-sport team loses a game, it feels like a personal failure.  IDE 3 I feel a sense of ownership for my favorite e-sport team rather than being just a fan of the team. | 0.829  0.833  0.864 | 4.06  4.63  4.07 | 1.699  1.692  1.827 | (Jeong et al., 2021) |
| Future Watching Intention | INT 1 I intend to watch e-sport game online when it becomes available.  INT 2 I will make an effort to watch e-sport game online when it becomes available. | 0.860  0.799 | 5.69  5.54 | 1.275  1.450 | (Leung & Chen, 2017) |

Note. SFL: Standardized factor loadings; M: Mean; SD: Standard deviation.
